# Ectopic Overexpression of *SlHsfA3*, a Heat Stress Transcription Factor from Tomato, Confers Increased Thermotolerance and Salt Hypersensitivity in Germination in Transgenic *Arabidopsis*

**DOI:** 10.1371/journal.pone.0054880

**Published:** 2013-01-22

**Authors:** Zhenjun Li, Lili Zhang, Aoxue Wang, Xiangyang Xu, Jingfu Li

**Affiliations:** 1 College of Horticulture, Northeast Agricultural University, Harbin, Heilongjiang, China; 2 College of life science, Northeast Forestry University, Harbin, Heilongjiang, China; 3 Key Laboratory of Biology and Genetic Improvement of Horticultural Crops (Northeast Region), Ministry of Agriculture, Harbin, Heilongjiang, China; Iwate University, Japan

## Abstract

Plant heat stress transcription factors (Hsfs) are the critical components involved in mediating responses to various environmental stressors. However, the detailed roles of many plant Hsfs are far from fully understood. In this study, an Hsf (*SlHsfA3*) was isolated from the cultivated tomato (*Solanum lycopersicum, Sl*) and functionally characterized at the genetic and developmental levels. The nucleus-localized SlHsfA3 was basally and ubiquitously expressed in different plant organs. The expression of *SlHsfA3* was induced dramatically by heat stress, moderately by high salinity, and slightly by drought, but was not induced by abscisic acid (ABA). The ectopic overexpression of *SlHsfA3* conferred increased thermotolerance and late flowering phenotype to transgenic *Arabidopsis* plants. Moreover, SlHsfA3 played a negative role in controlling seed germination under salt stress. RNA-sequencing data demonstrated that a number of heat shock proteins (Hsps) and stress-associated genes were induced in *Arabidopsis* plants overexpressing *SlHsfA3*. A gel shift experiment and transient expression assays in *Nicotiana benthamiana* leaves demonstrated that SlHsfA3 directly activates the expression of *SlHsp26.1-P* and *SlHsp21.5-ER*. Taken together, our results suggest that SlHsfA3 behaves as a typical Hsf to contribute to plant thermotolerance. The late flowering and seed germination phenotypes and the RNA-seq data derived from *SlHsfA3* overexpression lines lend more credence to the hypothesis that plant Hsfs participate in diverse physiological and biochemical processes related to adverse conditions.

## Introduction

Plant heat stress transcription factors (Hsfs) are the critical regulators of the intricate matrix mediating the expression of genes responsive to a wide range of stressors [1], [2], [3], [4], [5], [6]. They specifically bind to the palindromic heat shock elements (HSEs: 5′-AGAAnnTTCT-3′) conserved in promoters of heat stress (HS)-inducible genes of all eukaryotes [7], [8], [9], [10]. Among all the genes activated under HS, the heat shock protein (Hsp) genes are ubiquitously and rapidly induced. The protein products of the Hsp genes protect plants from damage by functioning as molecular chaperons to assist in protein folding, assembly, translocation, and membrane stabilization [11], [12], [13], [14], [15], [16]. Furthermore, almost all members of the plant Hsf family share common structural properties, including a highly conserved DNA-binding domain (DBD), an oligomerization domain (HR-A/B region), a nuclear localization signal (NLS), and, in most cases, a C-terminal activation domain characterized by short peptide motifs (AHA motifs) [3], [4], [5], [17]. Based on the peculiarities of their oligomerization domains, plant Hsfs are grouped into three classes (class A, B, and C). To date, 21, 52, 24 and 25 representatives have been identified in *Arabidopsis*, soybean, tomato and rice, respectively [3], [17].

To date, an ever-increasing body of studies about plant Hsfs has focused mostly on their roles in HS response. For example, HsfA1a plays an irreplaceable role as a master regulator for induced thermotolerance in tomato. Transgenic tomatoes overexpressing *HsfA1a* showed remarkable tolerance under severe high temperature treatment, whereas the co-suppression lines with knock-down of HsfA1a expression were very heat-sensitive, sustaining serious damage at exposure to 45°C for 1 h [18]. In the complex family of the plant Hsfs, HsfA2 has attracted more attention than others. HsfA2 accumulates to quite high levels and becomes the dominant Hsf under prolonged HS in both tomato and *Arabidopsis* [19], [20], [21]. Basal and acquired thermotolerance were remarkably enhanced in high-level *AtHsfA2*-overexpressing transgenic lines. However, the dominant negative mutants of *AtHsfA2* exhibited reduced thermotolerance [22]. AtHsfA2 also has been regarded as a key factor in sustaining the expression of Hsp genes and extending the duration of acquired thermotolerance in *Arabidopsis* [23]. In the *AtHsfA2*-overexpressing *Arabidopsis* plants, a number of HS-associated genes were highly induced and more than half of those genes were strongly repressed in the *AtHsfA2* knockout plants [20]. SlHsfA2 may be directly involved in the activation of protection mechanisms in the tomato anther during HS [24]. Furthermore, the thermotolerance of plants overexpressing *AtHsfA3* was elevated, and that of *hsfA3* T-DNA insertion mutants was decreased [25], [26]. The function of HsfA3 from *Lycopersicon peruvianum* (*LpHsfA3*) was proved to be similar to that of other tomato Hsfs in tomato cell cultures and yeast cells [27]. However, the target genes of both tomato and *Arabidopsis* HsfA3, and their contribution to plant HS response, have been rarely reported until now.

In addition to these studies, some evidence shows that several Hsfs could fulfill specific functions. In tomato, class B Hsfs, lacking the capacity to activate transcription, could serve as coactivators cooperating with class A Hsfs to synergistically activate the expression of downstream reporter genes. Moreover, tomato HsfB1 also cooperates with other activators in a similar manner to control housekeeping gene expression [28]. Surprisingly, soybean GmHSFB1 was reported earlier to be potentially involved in the inhibition of promoter activity in transient reporter assays [29], [30]. The functional characterization of a class C Hsf has been reported recently in *Oryza sativa* (Os). OsHsfC1b serves as a regulator of salt stress response and affects plant growth under non-stress conditions [31]. Moreover, previous studies have indicated that HsfA4 has a negative correlation with the levels of ascorbate peroxidase 1 (APX1) and may function as an anti-apoptotic factor in plants [32], [33], [34]. In both tomato and *Arabidopsis*, HsfA5 interacts physically with HsfA4 to form hetero-oligomers; in this way, HsfA5 acts as a specific repressor of HsfA4, which is a potent activator of heat stress gene expression [35]. As a specialized Hsf in plants, HsfA9 not only contributes to basal thermotolerance during the early hours of seed germination, it also plays a crucial role in embryogenesis and seed maturation in the absence of environmental stress. There is also evidence showing that HsfA9 works downstream of ABI3, a seed-specific transcription factor in the ABA signaling pathway [36], [37], [38], [39]. Plant Hsfs also could work with other Hsfs or non-Hsf factors to complete their mission [19], [40]. These special properties of plant Hsfs have deepened our understanding of the diversity of Hsf function and the high complexity of the Hsf family.

In addition to the increased thermotolerance conferred by most plant Hsfs studied, tolerance to other abiotic stresses can also be elevated as a consequence of overexpression of several Hsfs. *AtHsfA2*-overexpressing transgenic plants showed enhanced tolerance to both heat and salt/osmotic stress [22]. Recently, it has also been reported that the expression of *AtHsfA2* could be significantly induced under several stress conditions, including exposure to hydrogen peroxide, and it acts as a key regulator in the construction of increased tolerance to combined environmental stressors [20]. Constitutive overexpression of the seed-specific HsfA9 from sunflower is sufficient to confer tolerance to severe dehydration [41]. Transgenic *Arabidopsis* overexpressing *OsHsfA2e* exhibited tolerance to high-salinity stress [42]. Landmark studies have demonstrated that *AtHsfA3* works directly downstream of *AtDREB2A* and *AtDREB2C*, which are important transcription factors involved in plant responses to drought and salt stress. All of these findings suggest the possible involvement of *AtHsfA3* in osmotic stress response and tolerance [25], [26], [43], [44].

Inhibition of growth and/or development is generally observed when plants are exposed to adverse environmental conditions. Several plant Hsfs, including AtHsfA2, OsHsfA2e, AtHsfA3, and BhHsf1, have been proved to be involved in growth retardation [22], [25], [42], [45].

Seed germination is antagonistically controlled by the phytohormones gibberellic acid (GA) and abscisic acid (ABA) [46], [47]. It is widely acknowledged that GA promotes seed germination, whereas ABA blocks germination. GA-ABA crosstalk plays a central role in the regulation of seed germination under high salinity conditions [48]. It has been reported that GA promotes seed germination by enhancing the proteasome-mediated degradation of RGL2, a key DELLA factor repressing germination. Meanwhile, LEAFY PETIOLE (LEP) functions as a positive regulator of GA-induced germination acting downstream or independently of RGL2 [46], [49].

The aim of our research is to estimate the candidacy of *SlHsfA3* for the genetic manipulation of heat and other stress tolerance of important commercial crops. In this study, we characterized the function of SlHsfA3, mainly from the genetic and developmental perspectives, using transgenic approaches.

## Materials and Methods

### Plant material, growth conditions, and stress treatments

Tomato cv. 04078 was used for the isolation of *SlHsfA3*. Seeds of 04078 were obtained from the The World Vegetable Center (AVRDC). Tomato seedlings were grown in a growth chamber maintained under 16 h of light (200 μE m^−2^ s^−1^) at 28°C and 8 h of dark at 18°C. *Nicotiana benthamiana* was grown under these same conditions.

*Arabidopsis thaliana* ecotype Col-0 was used as the wild-type. *Arabidopsis* seeds were surface sterilized for 15 min in 10% bleach, washed five times with sterile water, and plated on half-strength Murashige and Skoog (MS) medium containing 0.8% (w/v) Bacto Agar [50]. Sterilized seeds were stratified at 4°C for 2 d in darkness and then transferred to a phytotrone set at 22°C with a 16-h-light/8-h-dark photoperiod.

For the expression pattern analysis of *SlHsfA3* in tomato, seedlings of 04078 were prepared and the stress treatments were performed as follows. For heat stress, 4-week-old tomato seedlings were exposed to 42°C for 12 h in a climate chamber, and the aerial parts were harvested at each time point. The spatial expression profile of *SlHsfA3* was evaluated by collecting different organs from 10 4-week-old tomato seedlings grown under normal conditions. For salt and ABA treatments, 250 mM NaCl and 100 μM ABA were each supplied to excised 3-week-old tomato plants through their cut stems [51]. Excised plants were placed in 2-ml tubes containing 1.5 ml of liquid MS medium with each elicitor. Control tubes contained equal amounts of liquid MS medium alone. Stock solution of ABA (mixed isomers; Sigma) was in methanol. Wound treatment was performed as described by Constabel *et* *al*. (1995) [52]. For drought stress, the aerial parts of 3-week-old tomato seedlings were detached and placed on a dry filter paper. At each time point of all stress treatments described, 10 independent tomato plants were pooled for sampling and frozen in liquid nitrogen for the subsequent qRT-PCR analysis.

### Molecular cloning and sequence analysis

In order to obtain the exact *SlHsfA3* sequence of our own material, we retrieved the full-length *SlHsfA3* cDNA sequence (homolog of *LpHsfA3*), using the rapid amplification of cDNA ends (RACE) method (Takara, Japan). The nucleotide sequence encoding DNA-binding domain of LpHsfA3 was used to design the gene-specific primers. The PCR products were cloned into the pGEM-T cloning vector (Promega, USA) and subjected to sequencing. The cDNA sequence was then submitted to NCBI. Amino acid sequence alignment was performed using ClustalX 2.0 and DNAMAN programs. Heat shock elements (HSEs) were drawn according to the nomenclature described by Nover *et* *al*. (2001) [3].

### DNA constructs and plant transformation

The *SlHsfA3* promoter region was PCR amplified with the primers *SlHsfA3_pro_*-F, 5′-CCCAAGCTTGAGTAGTGCGAACAAATAC-3′, and *SlHsfA3_pro_*-R, 5′-CGCGGA TCCATCATACAAAGAGATTTG-3′. The 1.3-kb PCR product was cloned into the *Hind*III-*Bam*HI sites of the binary vector pCAMBIA1391-Z to generate the *SlHsfA3_pro_* **:** *GUS* construct. *SlHsfA3* cDNA was PCR amplified from the reverse transcription product with primers 5′-CACCATGAACCCATTTGATAAAAAACAA GAATC-3′ and 5′-CTAGAAACTATCATTCTTGGGCTGG-3′. The PCR product was first cloned using a pENTR Directional TOPO cloning kit (Invitrogen, USA) and then recombined with the plant binary vector pGWB6 [53] to generate the *35S_pro_* **:** *sGFP-SlHsfA3* construct. Based on this vector, primers 5′-CATGCCATGG TGATGGTGAGCAAGGG-3′ and 5′-GGAAGATCTGATCTAGTAACATAGATGAC ACC-3′ were used to amplify the *sGFP-SlHsfA3-NOS terminator* fragment which was subsequently cloned into the *Nco*I-*Bgl*II sites of the *SlHsfA3_pro_* **:** *GUS* construct to generate *SlHsfA3_pro_*:*sGFP-SlHsfA3* construct. The binary vector pBI121 was first digested with *Xba*I and *Sac*I to remove the *GUS* gene. *SlHsfA3* cDNA was PCR amplified with primers 5′-CTAGTCTAGAATGAACCCATTTGATAAAAAACAAG AATC-3′ and 5′-CGAGCTCCTAGAAACTATCATTCTTGGGCTGG-3′. The PCR product was cloned into the *Xba*I-*Sac*I sites of the pBI121 fragment to generate the *35S_pro_* **:** *SlHsfA3* construct. The above constructs were then transformed into the *Agrobacterium tumefaciens* strain GV3101 (pMP90), which was used for transformation of *Arabidopsis* plants (Col-0) by the floral dip method [54].

### Gene expression analysis

For qRT-PCR analysis, total RNA was isolated from plant materials with Trizol (Invitrogen, USA) according to the manufacturer's instructions. Poly (dT) cDNA was prepared from 2 μg of total RNA with M-MLV reverse transcriptase (Promega, USA) and quantified with a cycler apparatus (Bio-Rad) with the SYBR *Premix Ex Taq* (Takara, Japan) according to the manufacturer's instructions. PCR was performed in 96-well optical reaction plates heated for 30 s at 95°C to activate hot start Taq DNA polymerase, followed by 40 cycles of denaturation for 5 s at 95°C, annealing for 30 s at 60°C, and extension for 20 s at 72°C. Expression levels of target genes were normalized to those of *ACTIN2*, *ACTIN1* and *ACTIN7* for tomato, tobacco, and *Arabidopsis*, respectively [55], [56], [57]. The 2^−ΔCt^ method was used for the analysis and visualization of our qRT-PCR data. The statistical significance was evaluated by Student's *t*-test. Primers used to quantify gene expression levels are listed in Table S2.

For semi-quantitative RT-PCR assay, RNA extraction and reverse transcription reaction were performed as mentioned above. The PCR conditions for amplification of *SlHsfA3* were as follows: 5 min at 94°C, followed by 32 cycles of 15 s at 94°C, 30 s at 60°C, 20 s at 72°C. The same conditions were used in the amplification of *ACTIN7* of *Arabidopsis*, except that the number of PCR cycles was decreased to 24.

### GUS histochemical analysis

Plants from four independent transgenic *Arabidopsis* lines, all containing a single copy of *SlHsfA3_pro_* **:** *GUS* construct, were used for histochemical staining of GUS activity, which was detected according to the method described by Jefferson *et* *al* [58]. Whole seedlings or different tissues were soaked in the GUS staining buffer (1 mM X-glucuronide in 100 mM sodium phosphate, pH 7.2, 0.5 mM ferricyanide, 0.5 mM ferrocyanide, and 0.1% Triton X-100), subjected briefly to a vacuum, and incubated at 37°C in the dark from 3 h to overnight depending on the experimental requirement. After being washed with 70% ethanol several times, plants or tissues were photographed using the Leica DFC 490 stereomicroscope and Leica DM5000B microscope. For HS experiment, the 6-d-old transgenic seedlings were exposed to 37°C for 8 h and were allowed to recover at growth conditions for 3 h before histochemical staining. Images were processed with Adobe Photoshop CS 8.0.

### GFP visualization

T_3_ generation of transgenic plants harboring a *SlHsfA3_pro_*:*sGFP-SlHsfA3* construct were used for the analysis of subcellular localization of SlHsfA3-GFP fusion proteins (modified from Yoshida *et* *al*., 2008) [25]. Five-day-old seedlings grown on MS medium were observed using a Leica TCS SP5 confocal laser scanning microscope. Before microscopy, seedlings were briefly stained with 10 μg/mL propidium iodide for 5 min and washed once with water. The excitation wavelengths for propidium iodide and GFP were 488 nm and 561 nm, respectively, and emission was detected using wavelengths of 600 to 640 nm and 500 to 540 nm, respectively. Approximately 10 seedlings were examined, and three independent experiments were done, yielding similar results.

### Phenotypic analysis of transgenic *Arabidopsis* plants

Seedlings were grown on MS medium containing agar in Petri dishes. The Petri dishes were immersed in a water bath at different temperatures for heat tolerance assays. Eight-day-old transgenic and Col-0 plants were exposed to 43°C for 1 h for the basal thermotolerance assay and to 37°C for 1 h, 22°C for 3 h, and 47°C for 1 h for the acquired thermotolerance assay (modified from Zhu *et* *al*. 2009) [45]. About 50 plants of each genotype were used. These plants were then incubated at 22°C for 6 d before photographs were taken and the survival rates were calculated. The detailed morphology of plants at 0 or 4 d after HS treatment was observed using 4-d-old plants grown vertically.

For the germination assays under stress treatment, seeds of homozygous transgenic lines and Col-0 were placed on MS agar medium supplemented with NaCl of different concentrations. The percentage of germinated seeds was scored daily and photographs were taken 3 d after stratification. Germination was defined as a clear sign of the emergence of radicle tip and the germination results were calculated based on four independent experiments.

For *LEP* and *RGL2* expression assays, about 1000 surface sterilized seeds of each genotype were soaked in liquid MS medium, with or without 120 mM NaCl, and were harvested 16 h after stratification.

For the root length assay, seeds were first germinated and grown vertically on MS agar medium for 4 d, followed by transfer to fresh medium (in the absence or presence of NaCl) for vertical growth for another 4 d, after which root length was measured with a ruler and photographed. For the salt tolerance assay, seeds were sown on filter paper laid over the surface of MS agar medium. Two weeks later, salt stress treatment was performed by saturating the filter paper with 150 mM NaCl solution for 6 h.

### RNA-seq analysis

RNA-seq analysis was carried out using two independent transgenic lines (#3 and #6). Total RNA was isolated with Trizol reagent (Invitrogen, USA) from the aerial parts of 4-week-old seedlings of *35S_pro_* **:** *SlHsfA3* and Col-0 plants grown in parallel under unstressed conditions. Material from 20 plants of each genotype was pooled for RNA isolation. cDNA synthesis was performed according to the previously described method with some modification [59]. The newly synthesized double-strand cDNA was fractured into 300–500 bp fragments using an ultrasonic instrument (Fisher) and then purified with AMPure beads (Agencourt, USA). The sequencing library was prepared and PCR amplified using TruSeq^TM^ DNA Sample Prep Kit-Set A and TruSeq PE Cluster Kit, respectively (Illumina, USA). Sequencing was performed on the Illumina HiSeq 2000 platform. The number of sequencing reads generated from each sample was converted into RPKM (reads per kilobase of exon model per million mapped reads) [60]. The DEGseq package was used for identifying genes differentially expressed between two samples [61]. All changes in gene expression were statistically significant at Q-value <0.05 in both overexpression lines [62], [63]. RNA-seq data were submitted to NCBI and can be accessed under the GEO accession number GSE40388. Pathways and Gene Ontology (GO) analyses were performed using the Molecule Annotation System (MAS).

### Gel-shift (EMSA) assay

To construct a plasmid for the expression of recombinant SlHsfA3 protein in *E. coli*, the full-length cDNA fragment was amplified by PCR using primers 5′-CCGGAATT CATGAACCCATTTGATAAAA-3′ and 5′-ACGCGTCGACCTAGAAACTATCATT CTTG-3′ and cloned into the pMAL-c2 vector via *EcoR*I and *Sal*I restriction sites. The *MBP*-fused *SlHsfA3* construct was transformed into *E. coli* BL21 cells. The MBP-SlHsfA3 fusion protein was induced and purified according to the manufacturer's instructions. Oligonucleotide probes were synthesized and labeled with biotin at the 5′ end (Invitrogen, USA). Mutated probes were synthesized using 5′-AAAnnAAA-3′ to replace the typical form of 5′-GAAnnTTC-3′. Labeled probes and nonlabeled cold competitor probes were generated from dimerization. Electrophoretic mobility shift assay (EMSA) was performed using a LightShift Chemiluminescent EMSA kit (Thermo Scientific). Probe sequences are shown in Table S2.

### Transactivation of *SlHsp26.1-P* and *SlHsp21.5-ER* promoter activity by SlHsfA3 in *N. benthamiana* Leaves

The transient expression assays were performed in *N. benthamiana* leaves as previously described [64], [65]. The N-terminal fragment of *SlHsfA3* was PCR amplified using primers 5′-CTAGTCTAGAATGAACCCATTTGATAAAAAACAAG AATC-3′ and 5′-CGAGCTCCTATGCAAGGTCTTGAAA-3′. The PCR product was then cloned into *Xba*I-*Sac*I sites of pBI121 to generate *35S_pro_* **:** *SlHsfA3*Δ*C* construct. The *SlHsp26.1-P* promoter was amplified with the primer pairs 5′-CCGGAATTCGC ACAAGTACTCCTCAATC-3′ and 5′- CGCGGATCCCACAGAAAGTAGAAATCT TC-3′ and cloned into the *Eco*RI-*Bam*HI sites of the binary vector pCAMBIA1381Z-LUC which was previously modified by our laboratory staff to generate the reporter construct *SlHsp26.1-P_pro_*:*LUC*. Primer pairs 5′-CCGGAATTCG AGCAAGTTGACGTCTAG-3′ and 5′-CGCGGATCCACTATACACTGTAGTATTG -3′ were used for the *SlHsp21.5-ER* promoter amplification with the same restriction sites to generate the construct *SlHsp21.5-ER_pro_*:*LUC*. The full-length *SlHsfA3* effector construct was the above-described *35S_pro_* **:** *SlHsfA3*. *Agrobacterium*-mediated infiltration of *N. benthamiana* leaves was performed as described [66]. Infiltrated plants were incubated at 28°C for 72 h before CCD imaging. A low-light cooled CCD imaging apparatus (NightOWL II LB983 with indigo software) was used to capture the LUC image and to count luminescence intensity. The leaves were sprayed with 100 mM luciferin and were placed in darkness for 5 min before luminescence detection. Five independent determinations were assessed. Error bars represent SD. The experiments were repeated at least five times with similar results.

## Results

### Cloning and sequence analysis of *SlHsfA3*

**Figure 1:**
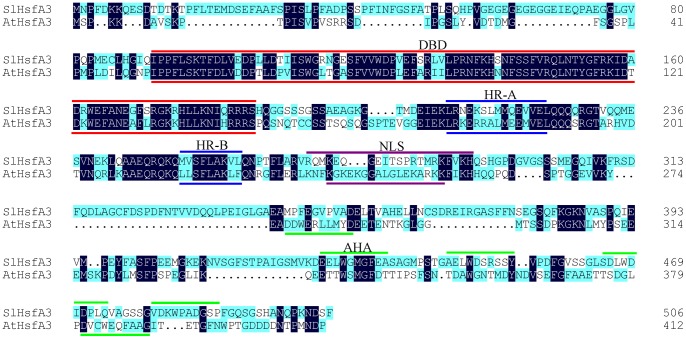
Alignment of HsfA3 proteins from tomato (*Solanum lycopersicum*; *Sl*) and *Arabidopsis* (*Arabidopsis thaliana*; *At*). The sequence alignment was performed using ClustalX 2.0 and DNAMAN software. Conserved amino acids in both proteins are highlighted in black and nonidentical residues are shaded in light blue. The dashes indicate gaps introduced for better alignment. The signature domains of both Hsfs are indicated by color bars: DNA-binding domain (DBD), red; heptad repeat pattern of hydrophobic amino acid residues (HR-A/B), dark blue; nuclear localization signal (NLS), purple; transcriptional activation domain (AHA), green.

The nucleotide sequence encoding the DNA-binding domain (DBD) of LpHsfA3 was used for designing gene-specific primers [27]. Subsequently, the corresponding full-length cDNA sequence containing 24 bp of 5′ UTR, 132 bp of 3′ UTR with poly A signal, and 1,521 bp of open reading frame (ORF) was cloned using RACE technology and designated as *SlHsfA3* (Accession No.GU120360), which shares nearly 97% sequence identity with *LpHsfA3*. The deduced amino acid sequence contains a highly conserved N-terminal DNA-binding domain (DBD), an oligomerization domain with the heptad pattern of hydrophobic amino acid residues (HR-A/B), and a putative nuclear localization signal (NLS) adjacent to the HR-A/B region. In addition, several features of class A Hsfs were found in SlHsfA3, including the 21-amino acid class-specific insertion extending HR-A/B region, and short peptide motifs enriched in aromatic and large hydrophobic amino acid residues embedded in an acidic surrounding (AHA motifs) (Fig. 1). Clustalx2.0 and DNAMAN were employed for generating sequence alignment between SlHsfA3 and AtHsfA3. The result revealed that they shared 36.87% identity over the whole amino acid sequence.

### Expression of *SlHsfA3* can be induced by multiple abiotic stresses

**Figure 2:**
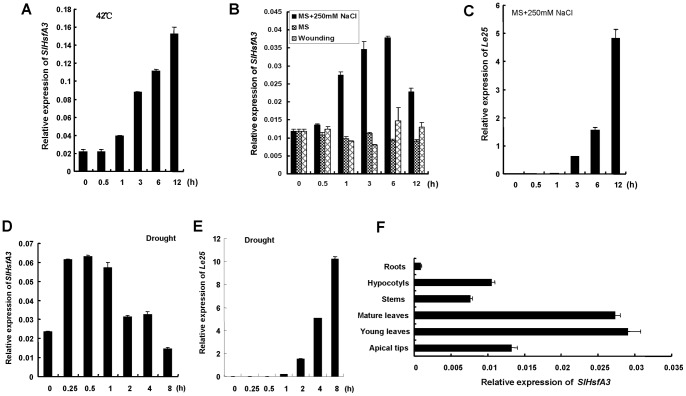
mRNA expression patterns of *SlHsfA3* in response to various abiotic stresses. (A) High temperature-induced expression pattern of *SlHsfA3* in tomato plants. Four-week-old tomato seedlings, grown under standard conditions, were placed in a climate chamber set at 42°C for 12 h. The aerial parts were harvested at the indicated times for RNA extraction and qRT-PCR analysis. Zero time samples were taken prior to treatment. Transcript levels of *SlHsfA3* were normalized to the *ACTIN2* expression. (B–E) High salinity and drought-induced expression patterns of *SlHsfA3* in tomato. Three-week-old tomato seedlings, grown under standard conditions, were used for stress treatments. The *SlHsfA3* mRNA levels were analyzed as in (A) and the *Le25* [67], [68] tomato gene was used as a positive control for high salinity and drought treatments. (F) Basal mRNA levels of *SlHsfA3* were quantified by qRT-PCR as described in (A) in different organs of tomato plants (n = 10). All stress treatments were initiated at the beginning of the 16 h light period (28°C) to ensure identical environmental conditions throughout the time course. Data shown are average and SD of triplicate reactions. Shown are representative data from one biological replicate and we conducted three biological replicates with similar results.

qRT-PCR analysis was carried out to investigate the expression patterns of the *SlHsfA3* gene under different abiotic stresses (Fig. 2). Under heat stress treatment (42°C), *SlHsfA3* expression was maintained at a relatively constant level within 0.5 h after the start of the treatment. Obviously increased transcripts of *SlHsfA3* were first detected at 3 h after treatment and the *SlHsfA3* transcript continued to accumulate thereafter and reached the peak level at 12 h (Fig. 2A). For high-salinity response, the *SlHsfA3* transcripts were moderately elevated and reached a maximum level at 6 h. After 12 h of NaCl application, the expression level of *SlHsfA3* was still higher than that in untreated plants. Moreover, *SlHsfA3* expression could not be induced by MS alone or by wounding (Fig. 2B). For drought stress, the *SlHsfA3* transcripts were only slightly accumulated during the 0.25–1 h period of treatment, followed by a return to levels found in untreated plants, and even a lower level at 8 h (Fig. 2D). No significant increase or decrease in the *SlHsfA3* expression level was observed in response to exogenous ABA treatment (Fig. S1A). The expression analysis of the *Le25* gene was used here as a positive control to indicate the effectiveness of different treatments (Fig. 2C, 2E, S1B) [67], [68]. These results demonstrate that *SlHsfA3* is involved in heat, salt, and possibly drought stress signaling pathways, and might serve as a master regulator in the plant abiotic stress response. Finally, the basal and organ-specific expression of *SlHsfA3* was studied. Based on the transcript levels, *SlHsfA3* was expressed ubiquitously in all organs analyzed, with higher levels of expression in young and old leaves compared with that in other parts (Fig. 2F).

**Figure 3:**
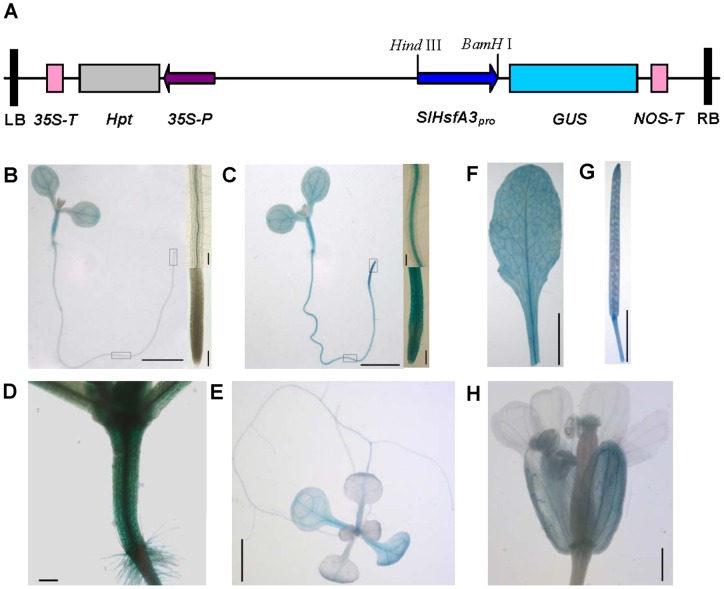
Histochemical analysis of *GUS* expression driven by *SlHsfA3* promoter in transgenic *Arabidopsis*. (A) Schematic diagram of construct used for agroinfiltration. (B–C) Six-day-old seedlings untreated (B) and heat stressed (C). For heat stress procedures, see Methods. Bars = 2 mm. The corresponding sections of vascular and meristematic tissues in root are boxed in (A) and shown by higher magnification on the right side. Bars = 100 μm. (D) Hypocotyl. Bar = 100 μm. (E) Twelve-day-old seedling. Bar = 2 mm. (F) Rosette leaves. Bar = 5 mm. (G) Mature siliques. Bar = 5 mm. (H) Flower. Bar = 500 μm.

To further investigate the temporal and spatial expression patterns of *SlHsfA3*, histochemical GUS staining of transgenic plants carrying a *SlHsfA3* promoter-GUS fusion construct was conducted. A 1.3-kb promoter region upstream of the *SlHsfA3* initiation codon was amplified from 04078 genomic DNA by PCR and used to drive the expression of the GUS reporter gene (Fig. 3A). The *SlHsfA3_pro_*:*GUS* cassette was stably transformed into *Arabidopsis*. Histochemical staining of transgenic plants exhibited GUS activity at almost all developmental stages. In 6-d-old seedlings grown under normal conditions, the GUS signal was relatively weak throughout the plants with almost undetectable GUS activity in roots (Fig. 3B). However, increased GUS staining was observed in plants that were previously heat stressed, mirroring the expression patterns of endogenous *SlHsfA3* in tomato. In particular, the roots presented very strong signals in response to high temperatures, and the vasculature was more strongly stained than were the epidermal tissues (Fig. 3C). It is worthy of note that significant GUS staining was present in hypocotyls (Fig. 3D). Moreover, only faint GUS signals could be detected in adult plants (Fig. 3E). Very strong GUS activity was observed in rosette leaves and mature siliques (Fig. 3F, G). In oral tissues, GUS signals were found in sepals but not in other parts (Fig. 3H). In summary, *SlHsfA3* promoter activity was distributed throughout most of the vegetative and reproductive organs.

### SlHsfA3-GFP fusion protein mainly targets the nuclei under heat stress

**Figure 4:**
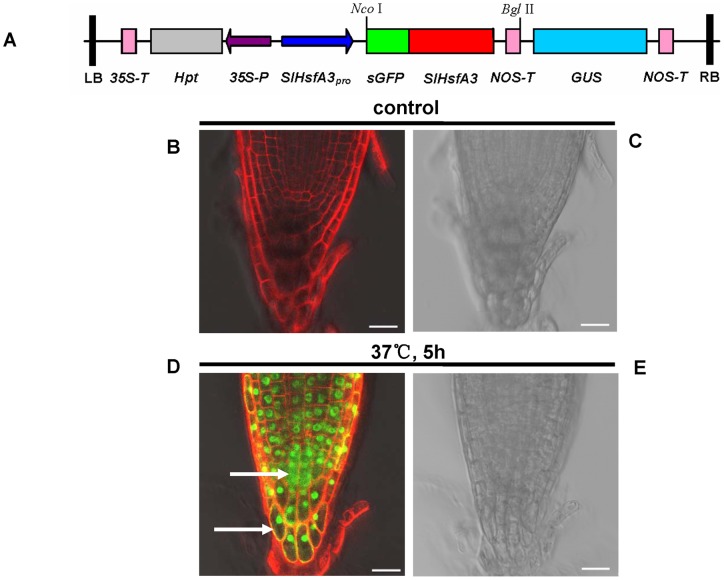
Subcellular localization of SlHsfA3-GFP fusion protein. (A) Schematic representation of construct used for *Arabidopsis* transformation. (B-E) The subcellular localization of SlHsfA3-GFP proteins in the root tips of transgenic *Arabidopsis* harboring *SlHsfA3pro:sGFP-SlHsfA3* construct. The root tips of 5-d-old seedlings were observed with a confocal microscope before (B and C) or immediately after (D and E) incubation at 37°C for 5 h. Bars  = 20 μm. The white arrows point to the cytoplasm and cell membrane, respectively. Propidium iodide staining was used to assess plasma membrane integrity (B and D).

Transcription factors commonly have a modular structure, and they have to transfer from the cytoplasm to the nucleus to activate genomic gene expression. The identified SlHsfA3 has a NLS motif that lies adjacent to the HR-B region (Fig. 1). To ascertain the subcellular localization of SlHsfA3 protein in *Arabidopsis*, we generated transgenic *Arabidopsis* carrying a sGFP-SlHsfA3 fusion protein construct under the control of *SlHsfA3* promoter, which is the same as that in the *SlHsfA3_pro_*:*GUS* construct (Fig. 4A). Root tips of 5-d-old T_3_ transgenic plants grown vertically were used for the observation of GFP fluorescence. Under normal conditions, almost no GFP signals were detected (Fig. 4B). In addition, detection of the GUS activity in the root tips of *SlHsfA3_pro_*:*GUS* transgenic plants showed similar results (Fig. 3B). An unknown regulatory mechanism may exist that limited the basal activity of *SlHsfA3* promoter in *Arabidopsis* root tips. For HS treatment, plants were first incubated at 37°C for 5 h and then GFP signals were examined immediately with confocal microscopy. Figure 4D shows that the SlHsfA3-GFP fusion protein mainly accumulated in nuclei. Moreover, a small amount of GFP fluorescence was detected in the cytoplasm of stem cells and in the membranes of root cap cells (marked with white arrows in Figure 4D). All of these results presented indicate that, under HS conditions, SlHsfA3 protein mainly targets the nuclei in *Arabidopsis*, which specifies the presence of NLS. This result is consistent with that derived from tomato cell cultures [27].

### Transgenic *Arabidopsis* overexpressing *SlHsfA3* showed increased thermotolerance and late flowering phenotypes

**Figure 5:**
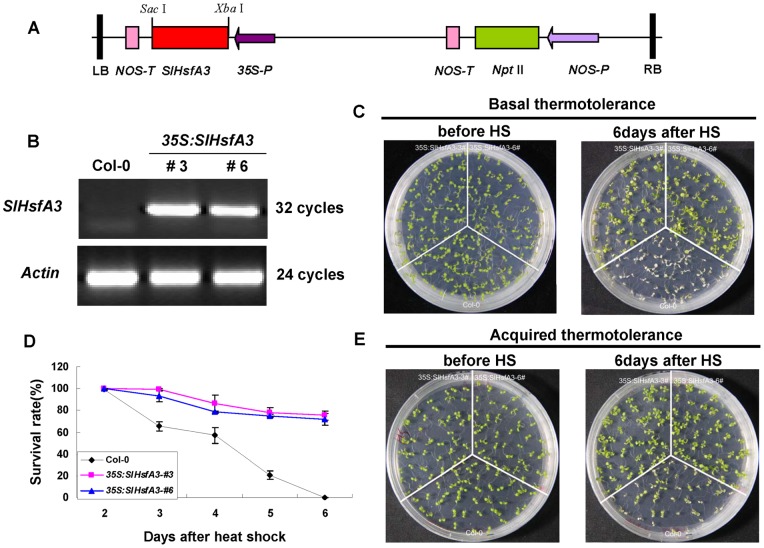
Thermotolerance of Col-0 and *35S:SlHsfA3* transgenic *Arabidopsis* plants. (A) Schematic representation of a construct used for *Arabidopsis* transformation of the *SlHsfA3* gene. (B) Relative expression of *SlHsfA3* in Col-0 and two T_3_ generation transgenic lines. Total RNA was extracted from 10-d-old seedlings, then analyzed by semi-quantitative RT-PCR. The *ACTIN7* gene was used as an internal control. (C) Comparison of basal thermotolerance among Col-0 and two OE lines. (D) Survival rates of Col-0 and two OE lines after HS at 43°C for 1 h. Seedlings that showed obvious etiolation appearance were considered to be dead. For each experiment, about 50 plants were used; values are means ± SD from three independent measurements. (E) Comparison of acquired thermotolerance among Col-0 and two OE lines.

To evaluate the effect of *SlHsfA3* on plant HS responses, we generated transgenic *Arabidopsis* plants ectopically overexpressing *SlHsfA3* under the control of the CaMV35S promoter and compared the acquired and basal thermotolerance of transgenic plants with those of Col-0. Two independent T_3_ transgenic lines (OE #3 and #6) were verified using semi-quantitative RT-PCR (Fig. 5A, B). Eight-day-old seedlings of each genotype, grown under standard culture conditions, were used for HS treatment. For basal thermotolerance test, all of the Col-0 seedlings were killed after being allowed to recover under standard conditions for 6 d, but nearly 80% seedlings of both *SlHsfA3*-overexpressing lines survived (Fig. 5C, D). After a conditioning pretreatment, 100% transgenic seedlings displayed the acquired thermotolerance but none of the Col-0 seedlings did (Fig. 5E). The above observation suggested that ectopic overexpression of *SlHsfA3* could confer increased basal and acquired thermotolerance to transgenic *Arabidopsis* plants.

**Figure 6:**
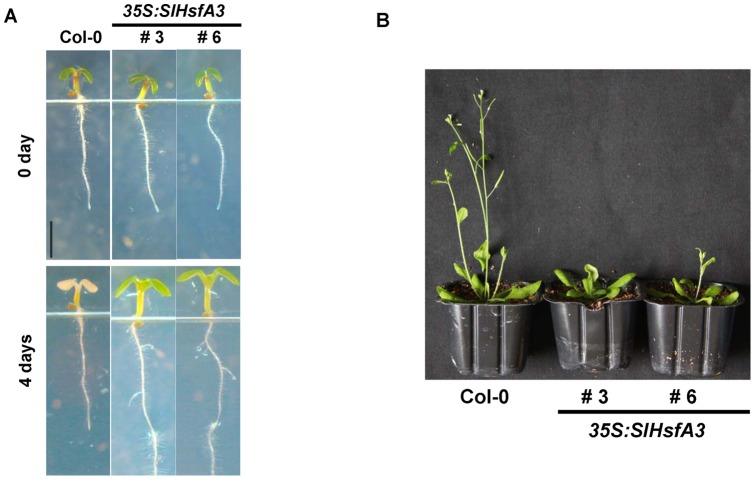
A detailed study of the enhanced thermotolerance brought by *SlHsfA3* and the flowering phenotypes of two *SlHsfA3* OE lines. (A) The detailed morphology of seedlings of Col-0 and two OE lines at 0 or 4 d after HS treatment. In this case, plants were grown vertically. Bar = 2.5 mm. (B) Col-0 and two *SlHsfA3* OE lines were grown under the same conditions as described in Methods. The image of four-week-old plants was taken on the same day.

In order to explore the detailed morphological differences between Col-0 and OE plants in thermotolerance, we monitored and compared the development of phenotype before and after HS treatment. No noticeable developmental differences could be observed between 4-d-old Col-0 and OE seedlings grown vertically under standard conditions (Fig. 6A). Morphological damage resulting from HS (43°C for 1 h) did not appear immediately, but became apparent after 4 d of recovery. As shown in Figure 6A, the Col-0 plants were severely injured and their cotyledons lost chlorophyll, whereas *SlHsfA3* OE plants stayed vigorous and did not show any observable injury syndrome. Moreover, the hypocotyls seemed to be less damaged than other parts of the Col-0 plants because they did not completely lose chlorophyll after 4 d of recovery. Concerning the challenge on roots, HS inhibited the growth of the main roots in the Col-0 plants. However, in the *SlHsfA3*-overexpressing plants, the main roots displayed relatively normal growth, accompanied by the emergence of lateral roots (Fig. 6A).

It is clear that defense against biotic stresses always occurs at the expense of growth [69], [70], [71]. This phenomenon could also be used to characterize the plants' responses to abiotic stresses. Previous studies have shown that growth retardation phenotypes are frequently detected in transgenic plants overexpressing abiotic stress-related genes [22], [45], [72]. In order to learn whether SlHsfA3 plays a part in regulating plant defense and growth, 11 T_3_ selected OE transgenic lines were monitored on a developmental scale and were compared with similarly monitored Col-0 plants. When grown in parallel, nearly all OE lines showed a late flowering phenotype, as represented by #3 and #6 (Fig. 6B). Except for the late flowering phenotype, no other remarkable differences on growth and development were observed.

### Overexpression of *SlHsfA3* in *Arabidopsis* results in seed germination sensitivity to salt stress

**Figure 7:**
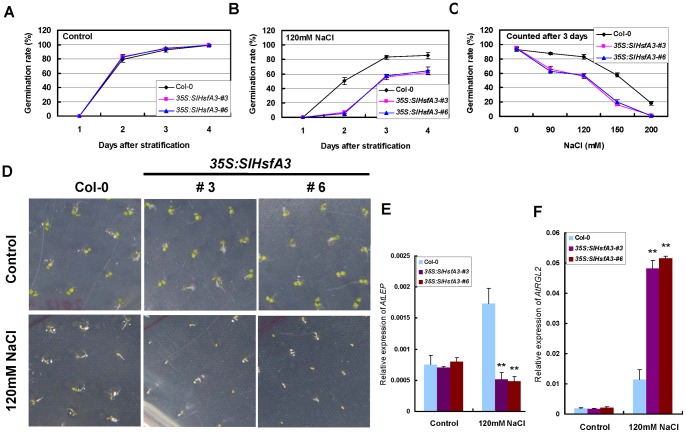
Germination assays of *SlHsfA3*-overexpressed plants. (A–C) Seed germination ratio of Col-0 and two OE lines in the absence and presence of NaCl. Seeds from different genotypes were germinated on MS agar plates (control, A) or supplemented with 120 mM NaCl (B) or with different concentrations of NaCl (C), respectively. Germination was defined as the obvious sign of radicle tip emergence and scored daily until the indicated day after incubation at 22°C. Data shown are average and SD of four independent experiments (each with 100 seeds for each genotype). (D) Increased germination sensitivity of *SlHsfA3* transgenic lines. Pictures were taken 3 d after stratification. (E–F) Expression of *AtLEP* and *AtRGL2* determined by qRT-PCR in germinating seeds of Col-0 and *SlHsfA3* transgenic lines treated with NaCl. All seeds were harvested 16 h after stratification. Error bars denote SD from three independent biological repeats. Asterisks denote Student's *t* test significance compared with Col-0 plants: **P<0.01.

Under normal growth conditions, there was no noticeable difference in seed germination of two selected transgenic lines (#3 and #6) compared with Col-0 (Fig. 7A). However, under high-salinity stress, the germination capability of seeds from the two *SlHsfA3*-overexpressing lines was much more reduced than that observed in the Col-0 seeds (Fig. 7B, C, D). For example, in the presence of 120 mM NaCl, half of the Col-0 seeds were successfully germinated on the second day after stratification, whereas the germination percentages of transgenic seeds were less than 10%. Even 3 and 4 d after being transferred to a phytotrone, the germination percentages of both transgenic lines were still significantly lower than those of Col-0 (Fig. 7B). A similar phenomenon was observed when seeds from different genotypes were germinated on MS agar medium supplemented with different concentrations of NaCl (Fig. 7C). This result excluded the possibility that the salt-hypersensitive phenotype in seed germination was due to a certain concentration of NaCl.

To evaluate how SlHsfA3 affects the salt sensitivity of transgenic plants during seed germination, we tested two well-known GA marker genes, *AtLEP* and *AtRGL2*, which were previously proved to be a positive and a negative regulator, respectively, of GA-induced germination [49], [73]. Seeds of different genotypes were immersed in liquid MS medium with or without NaCl (120 mM) supplementation, and incubated at 22°C for 16 h; they were then collected for the subsequent qRT-PCR analysis. We found that the expression levels of both *AtLEP* and *AtRGL2* were similar among all genotypes treated with MS medium alone. Supplemented with 120 mM NaCl, *AtLEP* transcripts were up-regulated more than two-fold in Col-0 seeds but were slightly down-regulated in seeds of transgenic *Arabidopsis* (Fig. 7E). Meanwhile, the induction of *AtRGL2* in transgenic seeds was much stronger than that in Col-0 seeds in response to NaCl (Fig. 7F). Based on the above results, we concluded that *SlHsfA3*-overexpressing transgenic plants were hypersensitive to salt stress during the seed germination stage. In addition, it is tempting to speculate that SlHsfA3 is perhaps involved in the GA signaling pathway in the control of seed germination.

Further phenotypic analysis of Col-0 and *SlHsfA3* OE lines in response to salt stress was performed at the post germination stage. Surprisingly, *SlHsfA3* OE lines exhibited neither salt-sensitive nor salt-resistant phenotypes at the post germination stage, as demonstrated by the root growth assay and qRT-PCR assay. As shown in Figure S2A, the growth of primary roots was nearly undifferentiated in the Col-0 plants compared with that of *SlHsfA3*-overexpressing lines when plants were grown vertically and exposed to different concentrations of NaCl. Statistical analysis of the root length measurements confirmed this result (Fig. S2B). In addition, the expression of several salt stress-related marker genes was analyzed in Col-0 and transgenic seedlings following NaCl exposure: *RD29A*, *RD29B*, *KIN1* [74], [75]. qRT-PCR analysis was performed using specific primers, and no significant differential induction for all of these marker genes was observed after 6 h of salt stress (Fig. S2C). The phenotypic analysis performed (as described above) confirmed that the increased germination sensitivity of transgenic *Arabidopsis* to salt stress is not associated with any change in the ability to survive salt stress at the post germination stage.

### RNA-seq analysis of aerial organs in transgenic *Arabidopsis*

In order to investigate the possible molecular mechanisms of SlHsfA3 function in plant growth and stress responses, we used the RNA-seq approach to identify the genes with altered expression levels in the *SlHsfA3* OE lines. Processing of RNA samples on the Illumina HiSeq 2000 system yielded more than 24 million reads, each 100 bp in length, encompassing 2.4 Gb of sequence data for each sample which was then mapped to the reference genome. Quantitative analysis of RNA-seq data identified substantial variation in expression profiles among different genotypes. Transcript abundance obtained from RNA-seq data was indicated as RPKMs, and therefore, up-regulated genes were determined by a greater than two-fold induction of normalized RPKMs in the comparison analyses (transgenic plants versus Col-0 plants) in both over-expression lines: Q-value <0.05 [62], [63]. In addition, the calculated correlation coefficient, based on the log-transformed RPKM values after eliminating genes with zero count in either of the two OE lines, was 0.9316, which indicated high correlation between the two OE lines (Fig. S3). These results demonstrate the very high reliability, reproducibility and quality of the raw data. Therefore, the mean of the results of the two independent OE lines are presented here in tables.

The statistical analysis identified a total of 181 differentially expressed genes between OE lines and Col-0 using Q-value <0.05 as a cutoff, among which 114 (63%) were up-regulated and 67 (37%) were down-regulated with fold changes higher than 2. With a more stringent screening Q-value of 0.001, table 1 contains 30 genes that were up-regulated more than three-fold in OE lines. These genes have been identified as participating in various aspects of plant physiological and biochemical activities, including stress response, metabolism, cellular transport, and so forth. Not surprisingly, Hsp genes dominated the list, and most of these were small Hsp genes. Hsps can function as molecular chaperones in stabilizing membranes, preventing non-native aggregation, and facilitating the subsequent refolding of non-native proteins [16], [76], [77], [78], [79]. Other stress responsive genes, such as *AtAPX2*, were indicated in table 1. *AtAPX2*, up-regulated more than 100-fold, encodes cytosolic ascorbate peroxidase, which scavenges reactive oxygen species [80]. Expression of *AtAPX2* could help plants maintain the activity of the antioxidant system which protects plants from oxidative damage due to adverse stresses [81]. *AtAPX2* was previously shown to be a direct target gene of AtHsfA2 [21]. *AtGolS1* encodes galactinol synthase, the rate-limiting enzyme of raffinose oligosaccharide synthesis. *AtGolS1* mainly functions in drought and high-salinity stress tolerance [82]. We also detected the up-regulated gene *AtMAF5* which encodes a negative regulator of flowering time [83], [84], [85]. The late flowering phenotype observed in *SlHsfA3* OE plants may be due, at least in part, to the up-regulation of *AtMAF5*. *AtEGY3* encodes an S2P-like putative metalloprotease which accumulates in response to heat, high light intensity, and hydrogen peroxide [20]. These results suggested that SlHsfA3 may regulate a group of stress-related genes, a notion consistent with the fact that the *SlHsfA3* OE lines exhibited better performance under heat stress.

**Table 1:** Up-regulated genes in *35S:SlHsfA3* transgenic plants (Q-value <0.001; fold change >3).

Gene locus	Annotation	Fold change	Q-value
***HSP***			
**AT4G27670**	Hsp25.3-P	87.1	7.97E-10
**AT4G10250**	Hsp22.0-ER	43.9	2.24E-05
AT5G37670	Hsp15.7-CI	23.6	4.01E-72
AT5G12030	Hsp17.7-CII	16.4	1.33E-149
AT3G46230	Hsp17.4-CI	11.2	1.74E-41
AT5G12020	Hsp17.6-CII	10.4	2.72E-39
AT1G53540	Hsp17.6C-CI	8.9	9.45E-13
AT2G29500	Hsp17.6B-CI	8.0	2.37E-41
AT2G32120	Hsp70T-2	6.1	3.55E-13
**AT4G21320**	Heat stress-associated 32 (Hsa32)	5.0	4.17E-09
AT3G12580	Hsp70, putative (Hsp70)	3.7	0.00E+00
***Transcription factor***			
**AT5G65080**	MADS-box family protein (MAF5)	15.4	2.04E-12
***Stress response***			
**AT3G09640**	Ascorbate peroxidase 2 (APX2)	128.0	4.01E-26
AT2G36750	UDP-glucosyl transferase family protein	10.9	5.08E-09
AT4G12400	Stress-inducible protein, putative	3.6	1.72E-48
***Metabolism***			
AT2G47180	Galactinol synthase (GolS1)	3.7	6.16E-164
AT1G47510	Inositol polyphosphate 5-phosphatase 11	3.6	7.96E-05
***Protein fate***			
**AT1G17870**	S2P-like putative metalloprotease (ATEGY3)	10.2	7.17E-187
AT4G21323	Subtilase family protein	9.8	4.10E-04
AT5G12110	Elongation factor 1B alpha-subunit 1	4.9	4.90E-109
AT5G48570	Peptidyl-prolyl cis–trans isomerase	3.4	3.96E-150
***Cellular transport***			
AT4G22505	Bifunctional inhibitor/lipid-transfer protein	4.5	1.94E-44
***Unclassified protein*** **/RNA**			
AT1G53480	*Arabidopsis* MTO1 Responding Down 1	62.3	2.64E-14
AT4G24415	miRNA824A	23.9	2.14E-04
AT1G53490	RING/U-box superfamily protein	14.2	5.91E-30
AT4G04410	Copia-like retrotransposon family	4.4	2.77E-15
AT4G16870	Copia-like retrotransposon family	3.9	2.65E-06
AT1G75750	Gibberellin-regulated protein 1 (GASA1)	3.4	1.12E-59
***Unknown protein***			
AT4G23493	Expressed protein	22.6	2.10E-10
AT3G10020	Expressed protein	5.8	5.73E-23

Fold change indicates the average of up-regulation in both lines compared with Col-0. Expressions of six bold-faced genes in this table and two other down-regulated genes (Table S1) were verified by qRT-PCR analysis (Fig. S5).

Genes that were down-regulated in the *SlHsfA3* OE lines (Table S1) also provided some useful information. Two of these genes were found to be related to pathogen responses. Thionin 2.1, a cysteine-rich protein, has the antibacterial and antifungal activities, properties that may be useful in the treatment of mammalian infectious diseases [86], [87]. *CESA4* encodes a cellulose synthase involved in secondary cell wall biosynthesis and functions as a negative factor on disease resistance in *Arabidopsis* [88]. Interestingly, the expression of *AtHsfA2*, which is a key regulator in HS response, was dampened in *SlHsfA3* OE lines (Table S1). To confirm this result, we performed an independent biological experiment to test the reduced expression of *AtHsfA2*. Eight-day-old seedlings of Col-0 and four OE lines (#2, #3, #6 and #10), grown on MS medium under standard conditions, were collected for RNA extraction and qRT-PCR analysis. As shown in Figure S4, the expression of *AtHsfA2* was clearly down-regulated obviously in all four OE lines. The underlying mechanism for this finding warrants further investigation.

To validate the expression profiles obtained by RNA-seq, we performed qRT-PCR analyses, using the same RNA samples as those used for RNA-seq, on eight genes randomly selected from Table 1 and Table S1. For all eight genes, the results agreed well with the RNA-seq data (Fig. S5). The complete gene expression profiling of our materials is provided in Table S3.

### SlHsfA3 directly regulates the expression of *SlHsp26.1-P* and *SlHsp21.5-ER*

**Figure 8:**
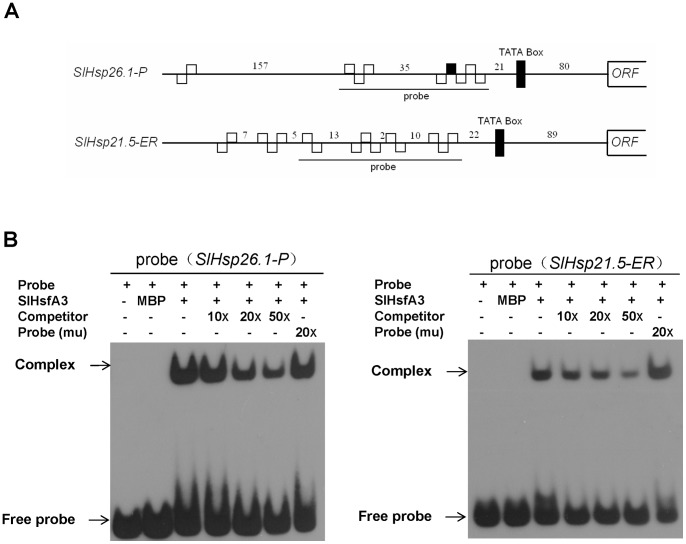
SlHsfA3 directly binds to the promoter regions of *SlHsp26.1-P* and *SlHsp21.5-ER*. (A) Illustration of the *SlHsp26.1-P* and *SlHsp21.5-ER* promoter regions showing the presence of HSEs DNA motifs. HSEs are drawn according to the nomenclature of Nover *et* *al*. (2001) [3]. Numbers in between indicate the distance in bp. The TATA box is also indicated in the schematic diagram. (B) EMSA assays showing that SlHsfA3 binds the HSEs motifs present in the *SlHsp26.1-P* and *SlHsp21.5-ER* promoters *in vitro*. SlHsfA3-MBP recombinant protein was expressed in *E. coli* cells. The corresponding probes were indicated in (A) and labeled with biotin. Competition for SlHsfA3 binding was performed with 10X, 20X and 50X cold unlabeled probes and 20X mutated probes. For details see Materials and methods.

The heat stress transcription factor SlHsfA3, like other members of tomato Hsfs, could serve as a key regulator in the HS response characterized by the expression of Hsp genes. As shown in Table 1, *Hsp25.3-P* and *Hsp22.0-ER* were the two most up-regulated sHsp genes. Based on this point, *SlHsp26.1-P* and *SlHsp21.5-ER*, two genes of tomato closely homologous to *Arabidopsis Hsp25.3-P* and *Hsp22.0-ER*, respectively, were screened using the BlAST server provided by sol genomic network (http://solgenomics.net). It is well known that Hsfs recognize and bind HSEs conserved in promoters of HS-inducible genes to function their way [3]. HSEs were commonly observed in the 5′ upstream region of the two genes (Fig. 8A), supporting a scenario that SlHsfA3 may directly associate with their promoters. HSEs were drawn according to the nomenclature of Scharf *et* *al*. (2001) [89]. A DNA EMSA was conducted to test the hypothesis that SlHsfA3 could directly bind the HSEs-containing DNA fragments present in the promoter regions of *SlHsp26.1-P* and *SlHsp21.5-ER*. Full-length SlHsfA3 protein was expressed as a maltose binding protein (MBP) fusion protein in *E. coli* and affinity purified. As shown in Figure 8B, the SlHsfA3-MBP fusion proteins were able to bind Biotin-labeled DNA probes containing several of the HSEs indicated in Figure 8A. Furthermore, this binding capacity could be effectively competed in a dose-dependent manner by the addition of excess amount of cold competitor probes, but not by the mutant form of probes (Fig. 8B). These results reveal that SlHsfA3 regulates *SlHsp26.1-P* and *SlHsp21.5-ER* expression through direct association with their promoters.

**Figure 9:**
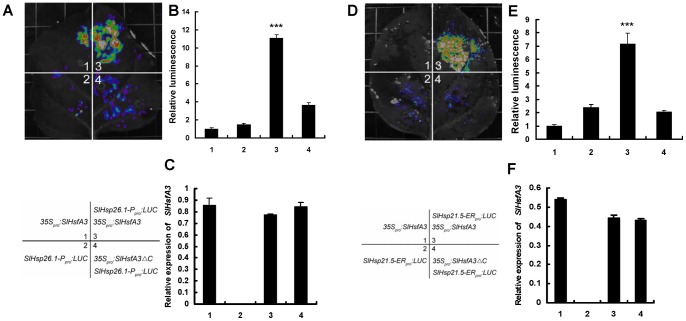
SlHsfA3 activates *SlHsp26.1-P* and *SlHsp21.5-ER* expression as revealed by transient assays of *N. benthamiana* leaves. (A) Transient expression assays showing that SlHsfA3 activates the expression of *SlHsp26.1-P*. Representative images of *N. benthamiana* leaves 72 h after infiltration are shown. The bottom panel indicates the infiltrated constructs and treatments. (B) Quantitative analysis of luminescence intensity in (A). Five independent determinations were assessed. Error bars represent SD. Asterisks denote Student's *t* test significance compared with control plants: ***P<0.001. (C) qRT-PCR analysis of *SlHsfA3* expression in the infiltrated leaf areas shown in (A). Total RNAs were extracted from leaves of *N. benthamiana* coinfiltrated with the constructs. Five independent determinations were assessed. Error bars represent SD. (D–F) Transient expression assays showing that SlHsfA3 activates the expression of *SlHsp21.5-ER*. Experiment procedures were the same as transient expression assays for *SlHsp26.1-P*.

Further evidence supporting this conclusion came from the well-established transient expression assay in *N. benthamiana* leaves. We verified the activation effect of SlHsfA3 on the expression of a reporter containing the *SlHsp26.1-P* or *SlHsp21.5-ER* promoter fused with the firefly luciferase gene (*LUC*). When the *SlHsp26.1-P_pro_*:*LUC* or *SlHsp21.5-ER_pro_*:*LUC* reporter or *35S_pro_* **:** *SlHsfA3* effector was infiltrated into *N. benthamiana*, the LUC activity could be barely detected. Co-infiltration of *SlHsp26.1-P_pro_*:*LUC* or *SlHsp21.5-ER_pro_*:*LUC* with the *35S_pro_* **:** *SlHsfA3* construct gave rise to an obvious induction in luminescence intensity (Fig. 9A, B, D, E), suggesting that SlHsfA3 can activate the above two reporter expressions in this transient expression assay. As a parallel, *35S_pro_* **:** *SlHsfA3*Δ*C*, in which the transcriptional activation domain was deleted, together with *SlHsp26.1-P_pro_*:*LUC* or *SlHsp21.5-ER_pro_*:*LUC* were co-infiltrated into *N. benthamiana* leaves. As shown in Figure 9, the activation effect of SlHsfA3ΔC on reporter expression was abolished. Taken together, our transient expression assays in *N. benthamiana* leaves indicated that SlHsfA3 directly activates *SlHsp26.1-P* and *SlHsp21.5-ER* expression in vivo.

The results obtained from EMSA and transient expression assays of *N. benthamiana* leaves helped us successfully identify two direct target genes of SlHsfA3. Due to the effectiveness and reliability of the two approaches, we expect to find out other bona fide target genes of SlHsfA3 in our follow-up work.

## Discussion

High temperature is one of the major limiting factors that could considerably reduce the yield of crops and impair their wider distribution. Therefore, exploring the complex molecular mechanism of plant response to HS has become a crucial subject of agricultural significance in recent years. Hsfs are the critical components that serve to regulate the expression of genes responsive to HS as transcription factors [2], [4], [5]. Hsfs have been well investigated in tomato and *Arabidopsis*. To date, a total of 24 predicted Hsf members have been identified in tomato [17]. One of these members, namely, SlHsfA3 is described here. This protein contains nearly all of the important signature domains of plant-specific Hsf proteins such as DBD, HR-A/B and AHAs (Fig. 1). The grouping of SlHsfA3 (class A) is due to an insertion of 21 amino acid residues between HR-A and HR-B [3]. The sequence alignment between SlHsfA3 and AtHsfA3 exhibited 36.87% amino acid identity.

The correlation between Hsfs and various abiotic stresses have been well established in previous studies [20], [22], [41], [42]. In this study, evidence from qRT-PCR analysis in tomato revealed that *SlHsfA3* could be strongly induced by high temperature, moderately by high salinity and slightly by drought, but was not induced by exogenous ABA treatment (Fig. 2, S1). It is possible that SlHsfA3 functions in an ABA-independent manner. The fact that *SlHsfA3_pro_*:*GUS* activity was boosted by HS and that seeds of *Arabidopsis* overexpressing *SlHsfA3* showed hypersensitivity to salt stress supports the link between SlHsfA3 and these abiotic stresses (Fig. 3, 7). In addition, as expected, *SlHsfA3* is capable of conferring increased thermotolerance to transgenic *Arabidopsis* (Fig. 5).

*SlHsfA3* could be induced, to different extents, by high salinity and drought as described above, whereas the *SlHsfA3*-overexpressing *Arabidopsis* plants did not exhibit any enhanced salt and dehydration tolerance compared with Col-0. This could be attributed to the inherent weak involvement of SlHsfA3 in these two signaling pathways and/or to the difference between species that limits the functioning of SlHsfA3.

To better understand the function of SlHsfA3, we used the Illumina Hiseq 2000 system to conduct RNA-seq experiments because of its superiority over the traditional microarray methods [90], [91], [92]. RNA-seq reports a larger dynamic range of expression levels than do microarray hybridizations. The gene expression comparison using our RNA-seq data confers the identification of a robust set of heat-responsive genes that could be used to advance us toward deciphering the high temperature regulatory networks. For example, the expression of several Hsps genes, including *Hsp25.3-P*, *Hsp22.0-ER*, *Hsp17.4-CI* and *Hsp17.6-CII*, as well as other stress-related genes such as *AtAPX2* could be up-regulated by the introduction of SlHsfA3. Previous studies have shown that these stress-responsive genes could also be activated by other plant Hsfs [20], [22], [25], [45]. These findings all point to a probable functional redundancy of plant Hsfs. In addition, as mentioned in the introduction to this paper, AtHsfA2 was proved to be a key regulator of thermotolerance in plants. The reduced expression of *AtHsfA2* in *SlHsfA3* OE lines was found in our RNA-seq data (Table S1) and confirmed by independent qRT-PCR experiments (Fig. S4). An antagonistic effect may exist between the two transcription factors. Further molecular and biochemical studies are needed to testify this hypothesis.

Plant flowering is under the control of both environmental stimuli and endogenous cues. Several pathways affecting flowering, such as photoperiod, vernalization, and GA pathways, have been extensively reviewed [93], [94], [95]. In our study, bolting and flowering of transgenic plants were delayed by several days as compared with Col-0 plants (Fig. 6B), and no other differences in growth and development could be observed. It is intriguing that *AtMAF5*, a negative regulator of flowering time, was among the set of up-regulated genes derived from our RNA-seq data (Table 1). The up-regulation of *AtMAF5* is very likely to contribute to the delayed flowering of transgenic *Arabidopsis*. These results proposed a role for *SlHsfA3* in plant reproductive growth.

Seed germination, one of the key steps during seedling development, is the beginning of the life cycle for many higher plants. Under abiotic stresses, the delay of seed germination might be due, in a large part, to the complex crosstalk between phytohormones [96]. In our study, *SlHsfA3* over-expression transgenic plants were hypersensitive to salt stress during the germination stage. In addition, under salt stress, the induction levels of two key regulatory genes of seed germination in the GA signaling pathway, *AtLEP* and *AtRGL2*, were significantly altered in seeds of transgenic lines compared to the induction levels of these genes seen in Col-0 seeds (Fig. 7E, F). This result was well-matched with the salt-hypersensitivity phenotype. We speculate that SlHsfA3 might interact with the GA pathway in controlling seed germination in response to high salinity. However, further studies are necessary to clarify the role of SlHsfA3 in the GA signaling pathway.

During high-temperature stress, transcription of Hsp encoding genes was the most common event triggered by the major components Hsfs [5], [17]. Hsp genes encode proteins that act primarily as molecular chaperones responsible for the stabilization of proteins and membranes under stress conditions [16]. To make a preliminary identification of the direct target genes of SlHsfA3 and based on the RNA-seq data, we found out two tomato Hsp genes, *SlHsp26.1-P* and *SlHsp21.5-ER*, that were homologous to the two most up-regulated Hsp genes shown in Table 1. EMSA and transactivation assays were used to test our assumption that the two genes were directly activated by SlHsfA3. As revealed by EMSA assays, SlHsfA3 specifically binds to HSEs present in the promoters of *SlHsp26.1-P* and *SlHsp21.5-ER* (Fig. 8). Using the well-established transient assays of *N. benthamiana* leaves, we showed that SlHsfA3 indeed stimulates the activity of both *SlHsp26.1-P* and *SlHsp21.5-ER* promoters, each fused with a reporter (Fig. 9). Collectively, these results support the notion that *SlHsp26.1-P* and *SlHsp21.5-ER* function as the direct target genes of SlHsfA3. However, it is important to note that we tested only two tomato Hsp genes and we can not rule out the possibility that SlHsfA3 may directly activate other Hsp genes and non-Hsp genes involved in response to various environmental stresses.

In conclusion, we characterized *SlHsfA3* as a multi-stress responsive gene in tomato. *Arabidopsis* overexpressing *SlHsfA3* showed increased thermotolerance, a late flowering trait, and hypersensitivity to salt stress at the germination stage. Although the functional exploration of SlHsfA3 is far from complete, the data we present here is of value for genetic modification of many economically important crops.

## Supporting Information

Figure S1 **ABA-induced expression patterns of** ***SlHsfA3*** **in tomato.** (A) ABA-induced expression patterns of *SlHsfA3* in tomato plants. Three-week-old tomato seedlings were used for stress treatment. Treatment protocols are as described in Methods. The *SlHsfA3* mRNA levels were analyzed as described in Fig. 2 (A). (B) The expression of *Le25* was used as a positive control for ABA treatment. Error bars indicate SD of triplicate reactions. Three independent biological replicates were performed.
(TIFF)

Figure S2 **Effects of high salinity on root lengths of Col-0 and** ***SlHsfA3*** **transgenic lines at post germination stage.** (A) Representatives of Col-0 and two OE lines treated with different concentrations of salt stress. Seeds of each genotype were germinated and grown on MS medium for 4 d and then transferred to new MS medium containing 120 mM and 200 mM NaCl for another 4 d. (B) Measurements of primary root lengths of plants shown in (A). All values are average and SD (n = 10). (C) Expression patterns of salt stress-responsive genes in Col-0 and two OE lines in response to salt stress. The induction of *RD29A*, *RD29B* and *KIN1* were quantified by qRT-PCR analysis. *ACTIN7* were used for normalization. The presented data are average and SD of triplicate reactions and three independent biological repeats were conducted with similar results.
(TIFF)

Figure S3 **The R^2^ linear regression of two transgenic lines.** The R^2^ values were calculated using the R statistics package (http://www.r-project.org/) based on the log-transformed RPKM values derived from RNA-seq data.
(TIFF)

Figure S4 **qRT-PCR confirmation of** ***AtHsfA2*** **with reduced expression in** ***SlHsfA3*** **OE lines.** Eight-day-old seedlings of Col-0 and four *SlHsfA3* OE lines (#2, #3, #6, #10) grown on MS medium under standard conditions were collected for RNA extraction and the subsequent qRT-PCR assay. Error bars indicate SD of triplicate reactions.
(TIFF)

Figure S5 **qRT-PCR validation of 8 genes with altered expression in both OE lines.** The RNA sample used for validation was the same as that used in RNA-sequencing. The selected 8 genes were also indicated in Table 1 and Table S1. Error bars indicate SD of triplicate reactions.
(TIFF)

Table S1 **Down-regulated genes in** ***SlHsfA3*** **OE plants** (**Q-value <0.001; fold change >2**)**.** Fold change indicates the average of down-regulation in both lines compared with Col-0. Expressions of two bold-faced genes in this table were verified by qRT-PCR analysis (Fig. S5).
(DOC)

Table S2 **List of the primers used in qRT-PCR, semi-RT-PCR and EMSA.**
(DOC)

Table S3 **Transcription profiling of** ***Arabidopsis*** **plants overexpressing** ***SlHsfA3*** **.**
(XLS)
