# Supplementary figures and images for: Ectopic Overexpression of SlHsfA3, a Heat Stress Transcription Factor from Tomato, Confers Increased Thermotolerance and Salt Hypersensitivity in Germination in Transgenic Arabidopsis
Source: PLoS One. 2013 Jan 22;8(1):e54880. doi: 10.1371/journal.pone.0054880 (PMC3551807; doi:10.1371/journal.pone.0054880)

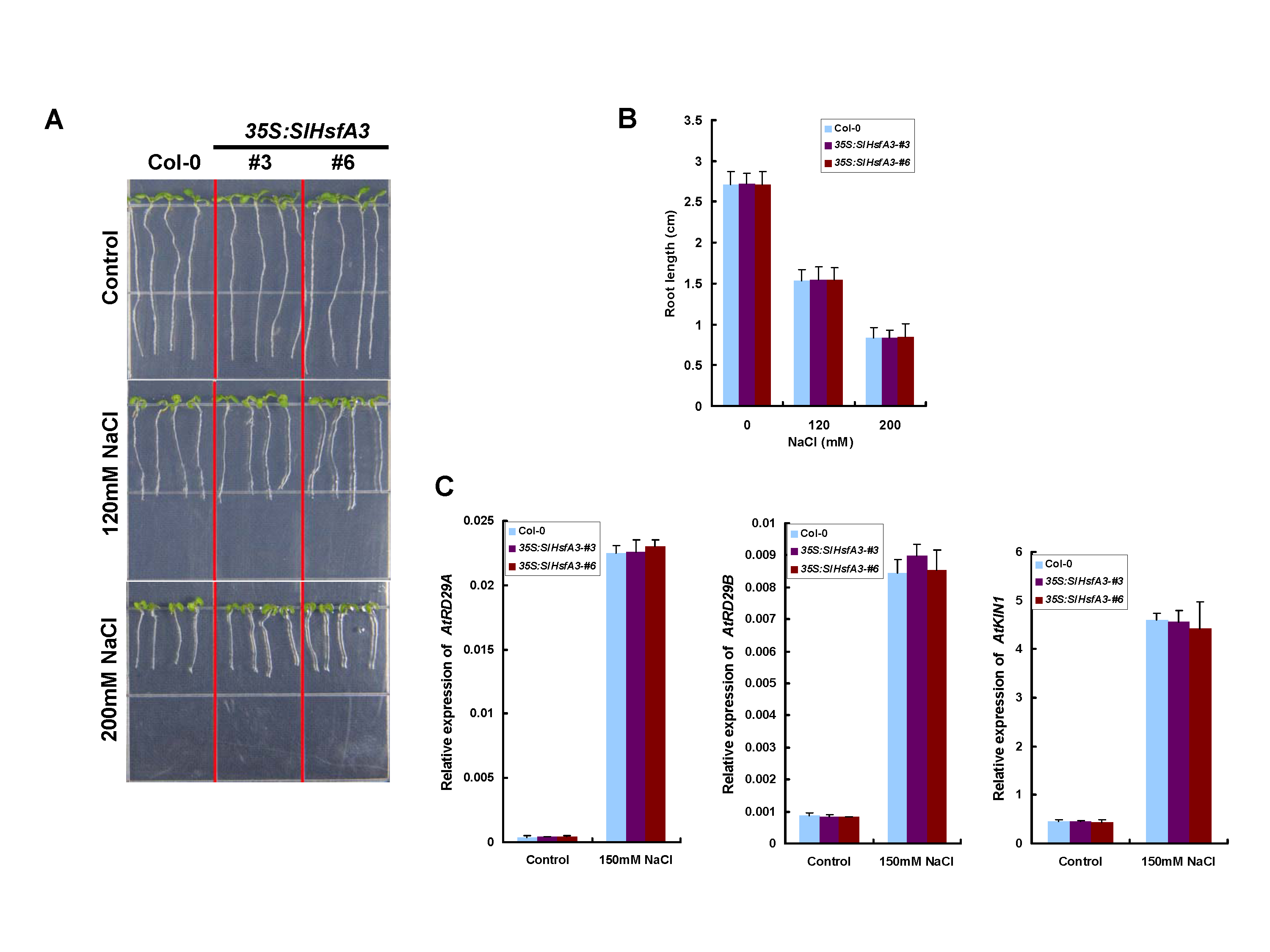

Supplement: Figure S2 — Effects of high salinity on root lengths of Col-0 and SlHsfA3 transgenic lines at post germination stage. (A) Representatives of Col-0 and two OE lines treated with different concentrations of salt stress. Seeds of each genotype were germinated and grown on MS medium for 4 d and then transferred to new MS medium containing 120 mM and 200 mM NaCl for another 4 d. (B) Measurements of primary root lengths of plants shown in (A). All values are average and SD (n = 10). (C) Expression patterns of salt stress-responsive genes in Col-0 and two OE lines in response to salt stress. The induction of RD29A, RD29B and KIN1 were quantified by qRT-PCR analysis. ACTIN7 were used for normalization. The presented data are average and SD of triplicate reactions and three independent biological repeats were conducted with similar results. (TIFF) [file pone.0054880.s002.tiff]

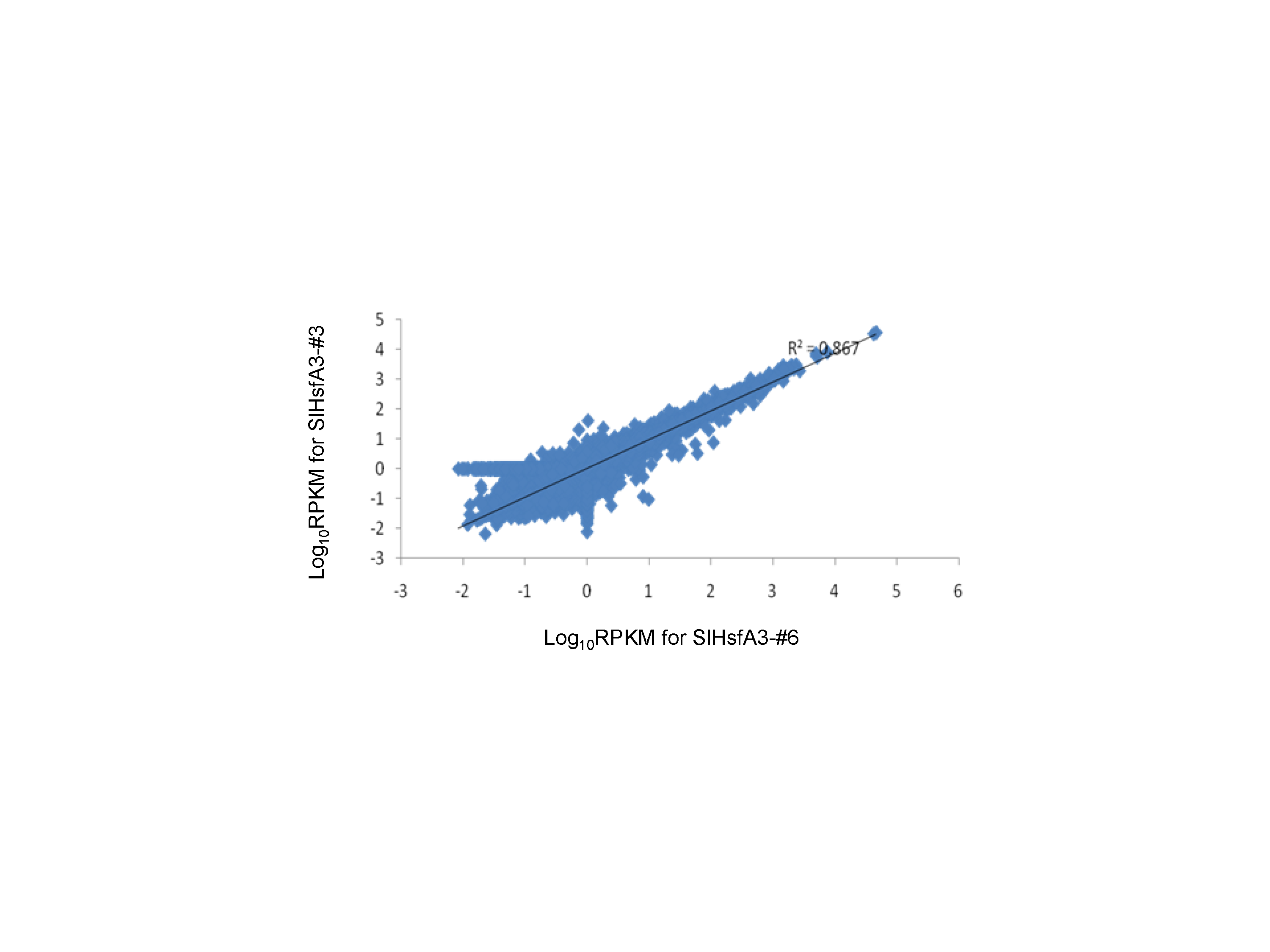

Supplement: Figure S3 — The R2 linear regression of two transgenic lines. The R2 values were calculated using the R statistics package (http://www.r-project.org/) based on the log-transformed RPKM values derived from RNA-seq data. (TIFF) [file pone.0054880.s003.tiff]
