# Supplementary material for: Ectopic Overexpression of SlHsfA3, a Heat Stress Transcription Factor from Tomato, Confers Increased Thermotolerance and Salt Hypersensitivity in Germination in Transgenic Arabidopsis
Source: PLoS One. 2013 Jan 22;8(1):e54880. doi: 10.1371/journal.pone.0054880 (PMC3551807; doi:10.1371/journal.pone.0054880)
Supplement: Table S1 — Down-regulated genes in SlHsfA3 OE plants (Q-value <0.001; fold change >2). Fold change indicates the average of down-regulation in both lines compared with Col-0. Expressions of two bold-faced genes in this table were verified by qRT-PCR analysis (Fig. S5). (DOC) [file pone.0054880.s006.doc]

**Table S1 Down-regulated genes in *SlHsfA3* OE plants (Q-value < 0.001; fold change > 2)**.

| ***Gene locus*** | ***Annotation*** | ***Fold change*** | ***Q-value*** |
| --- | --- | --- | --- |
| AT5G35935 | Transposable element gene | 64.92283 | 2.27E-07 |
| AT2G36255 | Defensin-like (DEFL) family protein | 32.46174 | 1.48E-05 |
| **AT3G56700** | Fatty acid reductase 6 (FAR6) | 30.43336 | 4.98E-23 |
| AT3G11500 | Small nuclear ribonucleoprotein family protein | 27.08849 | 1.32E-28 |
| AT1G22480 | Cupredoxin superfamily protein | 11.80427 | 3.13E-06 |
| AT1G34047 | Defensin-like (DEFL) family protein | 10.5159 | 6.15E-31 |
| AT4G11393 | Defensin-like (DEFL) family protein | 8.709236 | 4.90E-09 |
| AT1G63910 | MYB domain protein 103 | 6.121327 | 5.77E-05 |
| AT5G42180 | Peroxidase superfamily protein | 5.791771 | 2.05E-20 |
| AT1G72260 | Thionin 2.1 | 4.939865 | 1.00E-05 |
| AT5G62160 | Zinc transporter 12 precursor | 4.937131 | 7.11E-05 |
| AT5G03210 | Unknown protein | 4.504597 | 2.62E-06 |
| AT3G56980 | bHLH DNA-binding superfamily protein | 4.121007 | 3.44E-05 |
| **AT2G26150** | heat shock transcription factor A2 (HsfA2) | 3.674462 | 1.47E-14 |
| AT2G41240 | bHLH protein 100 | 3.614902 | 7.27E-08 |
| AT5G17420 | Cellulose synthase family protein | 3.541579 | 4.28E-18 |
| AT5G35480 | Unknown protein | 3.457958 | 4.29E-24 |
| AT1G66100 | Plant thionin | 3.263484 | 1.81E-13 |
| AT3G56970 | bHLH DNA-binding superfamily protein | 3.126049 | 1.95E-08 |
| AT4G12470 | Azelaic acid induced 1 (AZI1) | 3.084255 | 1.02E-11 |
| AT2G38080 | Laccase/Diphenol oxidase family protein | 2.857213 | 5.94E-08 |
| AT4G17470 | alpha/beta-Hydrolases superfamily protein | 2.813032 | 1.84E-04 |
| AT3G44450 | Unknown protein | 2.711735 | 8.75E-13 |
| AT1G47395 | Unknown protein | 2.700832 | 4.25E-07 |
| AT4G26530 | Aldolase superfamily protein | 2.700143 | 2.06E-57 |
| AT5G24420 | 6-phosphogluconolactonase 5 (PGL5) | 2.582042 | 3.50E-16 |
| AT1G47400 | Unknown protein | 2.447832 | 3.86E-04 |
| AT5G44030 | Cellulose synthase A4 (CESA4) | 2.438532 | 2.95E-15 |
| AT2G29310 | NAD(P)-binding Rossmann-fold superfamily protein | 2.402646 | 3.70E-05 |
| AT3G05880 | Low temperature and salt responsive protein family | 2.390712 | 5.60E-04 |
| AT3G11120 | Ribosomal protein L41 family | 2.238718 | 1.83E-05 |
| AT4G33865 | Ribosomal protein S14p/S29e family protein | 2.230453 | 1.73E-20 |

Fold change indicates the average of down-regulation in both lines compared with Col-0. Expressions of two bold-faced genes in this table were verified by qRT-PCR analysis (Fig. S5).
