# Supplementary material for: Ectopic Overexpression of SlHsfA3, a Heat Stress Transcription Factor from Tomato, Confers Increased Thermotolerance and Salt Hypersensitivity in Germination in Transgenic Arabidopsis
Source: PLoS One. 2013 Jan 22;8(1):e54880. doi: 10.1371/journal.pone.0054880 (PMC3551807; doi:10.1371/journal.pone.0054880)
Supplement: Table S2 — List of the primers used in qRT-PCR, semi-RT-PCR and EMSA. (DOC) [file pone.0054880.s007.doc]

**Table S2 List of the primers used in qRT-PCR, semi-RT-PCR and EMSA.**

| **Gene name** | **Primer sequence (5’ to 3’)** | **Note** |
| --- | --- | --- |
| *SlHsfA3* | F: AGATCCCTTGCAGGTAGCTG | qRT-PCR |
|  | R: TGATGGCAGTATCCCAATGG |  |
|  | F: TGAATTTGCTGCGTTTTCAC | semi-RT-PCR |
|  | R: GTAAACACTCCATAGGCTGC |  |
| *Le25* | F: AAGAGGATAGGGTTGCAGCG | qRT-PCR |
|  | R: GCAACTAGCTTCATTTCTCC |  |
| *AtHsp25.3-P* | F: TCAGCTCCCAGACAACTGTG | qRT-PCR |
|  | R: GTACAACAAACAGTACCTCC |  |
| *AtHsp22.0-ER* | F: AGCTTGAGAATGGTGTGCTC | qRT-PCR |
|  | R: CTCTCTTCAGACTCAGAAAG |  |
| *AtEGY3* | F: AGAAACACCCGCAAAAGACG | qRT-PCR |
|  | R: AGAACAATGCTTGTTTCTGC |  |
| *AtAPX2* | F: TCAGGATTCGAGGGTGCATG | qRT-PCR |
|  | R: AAGGCATCCTCATCTGCAGC |  |
| *AtMAF5* | F: GGAGCTTGTGAAGAACCTTC | qRT-PCR |
|  | R: TCAGCCGTTGATGATTGGTG |  |
| *AtHsa32* | F: AGCATCTGATGCGAAGTTGG | qRT-PCR |
|  | R: CACTAGATGTAGTGCGAGAG |  |
| *AtHsfA2* | F: GGAGGAACAATGTTTGGAGG | qRT-PCR |
|  | R: GACCGCAACAAGTAGATGTG |  |
| *AtFAR6* | F: TTGAGCACGAAGGGAAAGAG | qRT-PCR |
|  | R: GAGACCTGGAAGATGAACGT |  |
| *AtLEP* | F: TTGGTGGCTCTCAATCTTCG | qRT-PCR |
|  | R: GTCAGAAACCGGAGGCATTC |  |
| *AtRGL2* | F: AGCGCGTTTAAACAAGCGAG | qRT-PCR |
|  | R: TTCAGGCGAGTCATCTCTAC |  |
| *ACTIN2* | F: GGTGTGATGGTGGGTATGG | qRT-PCR |
|  | R: GCTGACAATTCCGTGCTC |  |
| *ACTIN1* | F: CATCAGGAAGGACTTGTACGG | qRT-PCR |
|  | R: GATGGACCTGACTCGTCATAC |  |
| *ACTIN7* | F: TCCATGAAACAACTTACAACTCCATCA | qRT-PCR and semi-RT-PCR |
|  | R: CATCGTACTCACTCTTTGAAATCCACA |  |
| *SlHsp26.1-P* | ATATACGATCTTTCGAGAAAGCTGGAGTTTAGCCTTGGCAATCACTTCACAGTTTTATCTATAACTTTCACGATTCCTCC | EMSA probe |
| *SlHsp21.5-ER* | TATAGTAAATTCCATTAACTTGTAAGGTGCTGGAAGTTTCAGAAGAAGTCTCTCAACGTTTTCGGAACTTTCAAGATTCT | EMSA probe |
